# Effectiveness of Distal Shoe Space Maintainers for First Permanent Molar Eruption: A Systematic Review

**DOI:** 10.3390/children12121642

**Published:** 2025-12-03

**Authors:** Laura Marqués-Martínez, Carla Esteve-Ferre, Lidia Galán-López, Juan Ignacio Aura-Tormos, Clara Guinot-Barona, Esther García-Miralles

**Affiliations:** 1 Dentistry Department, Medicine and Health Science Faculty, Catholic University of Valencia, 46001 Valencia, Spain; laura.marques@ucv.es (L.M.-M.); carla.esteve@ucv.es (C.E.-F.); lidia.galan@uv.es (L.G.-L.); 2 Stomatology Department, University of Valencia, 46010 Valencia, Spain; juan.aura@uv.es (J.I.A.-T.); m.esther.garcia@uv.es (E.G.-M.)

**Keywords:** distal shoe, space maintainer, premature tooth loss, eruption guidance, paediatric dentistry, systematic review

## Abstract

Background: Premature loss of the second primary molars can result in arch space loss and later malocclusion. Distal shoe space maintainers (SMs) are used to guide the eruption of the permanent first molars, although their clinical effectiveness remains under-evaluated. Objective: We aimed to assess the clinical effectiveness and complication profile of distal shoe SMs in paediatric patients following premature loss of second primary molars. Methods: A PRISMA-compliant systematic review was conducted, with protocol registered in PROSPERO (CRD420251101518). Searches in PubMed, Scopus, Web of Science, Embase, and Cochrane Library identified clinical studies evaluating distal shoe appliances in children aged ≤6 years. Risk of bias was assessed using ROBINS-I and JBI tools. Results: Six clinical studies comprising 67 patients were included. Overall eruption success was 95.5% (64/67), with minor complications occurring in 25.4% of cases. Modified designs demonstrated fewer complications, although sample size was limited. Due to heterogeneity of methods and outcomes across studies, results are reported as descriptive metrics rather than inferential estimates. Conclusions: Distal shoe space maintainers are effective in promoting eruption of the first permanent molar with an acceptable complication profile. Their success depends heavily on technical execution and case selection. Further prospective studies with larger cohorts are required to validate optimal procedural parameters.

## 1. Introduction

The premature loss of the second primary molars prior to eruption of first permanent molars affects 8–12% of children under six years [1]. This early tooth loss—most frequently caused by advanced caries, trauma, or molar–incisor hypomineralization—may trigger mesial drift in up to 73% of untreated cases [1], resulting in measurable arch length deficiency, midline deviation, and a predisposition to crowding and malocclusion requiring future orthodontic correction [1]. The clinical consequences of these sequelae underscore the importance of timely and biologically appropriate space-management strategies in paediatric patients.

Conventional fixed space maintainers, including band-and-loop and crown-and-loop designs, cannot be used before the eruption of the permanent first molar, as they require an erupted abutment tooth for anchorage [1]. Distal shoe appliances overcome this limitation via an intra-alveolar metal extension inserted beneath the gingival tissues and aligned with the eruption path of the unerupted molar [2]. This mechanical guidance facilitates a favourable eruption trajectory, helping prevent space loss and malocclusion. Success rates have been reported above 85% in several studies [3,4], although clinical outcomes remain technique-sensitive. The historical literature reports complications including gingival impingement, mucosal hypertrophy, cementation failures, and appliance instability [2].

More recent evidence provides updated perspectives on the clinical performance of distal shoe appliances. Rosenberg et al. reported frequent soft-tissue complications and cementation-related failures in a retrospective assessment of clinical outcomes [5]. Similarly, the evidence-based review by Sasa et al. underscores the need for standardisation in insertion depth, radiographic assessment, and tissue management protocols [6]. These observations are consistent with the recommendations of the American Academy of Pediatric Dentistry (AAPD), which emphasises that space maintenance must be determined by individualised evaluations of eruption timing, occlusal development, and expected tissue response [7].

In addition, the evolution of modified distal shoe designs—including reinforced acrylic bases, reduced-vertical extensions, and W-loop variations—reflects ongoing attempts to reduce complication rates and enhance stability [4,6]. These developments indicate that the distal shoe appliance continues to benefit from contemporary refinements rather than representing a static clinical technique.

Despite these advancements, existing evidence remains limited by small population sizes, heterogeneous methodologies, and a predominance of observational designs rather than prospective controlled trials. Accordingly, a contemporary synthesis of eruption effectiveness and complication-specific performance is necessary for clarifying the true clinical value of distal shoe appliances in paediatric space management.

Therefore, the aim of this systematic review was to evaluate the clinical effectiveness of distal shoe space maintainers in guiding the eruption of the first permanent molars following premature loss of second primary molars, and to assess complication rates, device performance characteristics, and technical determinants of success, in order to provide clinicians with practical, evidence-based recommendations for treatment planning.

## 2. Materials and Methods

### 2.1. Protocol and Registration

This systematic review adhered to the PRISMA 2020 reporting guidelines [7] and was prospectively registered in PROSPERO (CRD420251101518). The protocol defined the rationale, eligibility criteria, search strategy, and planned approach for risk of bias assessment and qualitative synthesis.

### 2.2. Elegibility Criteria

Population: paediatric patients aged ≤6 years with premature loss of a second primary molar prior to eruption of the permanent first molar.

Intervention: distal shoe space maintainers, including conventional designs and modifications.

Study design: randomised controlled trials, cohort studies, and case series with ≥3 patients.

Inclusion of single-patient case reports:

Due to the scarcity of available clinical evidence and high relevance of certain technical descriptions for this appliance, single case reports were included when they provided clinically relevant procedural or outcome data. These low-level evidence sources were analysed separately and weighted minimally to avoid bias.

Exclusion criteria:

- In vitro or animal studies;
- Narrative reviews;
- Case reports lacking clinical follow-up;
- Studies involving tooth types other than the second primary molar;
- Age > 6 years.

### 2.3. Information Sources and Search Strategy

A comprehensive literature search was performed in PubMed, Scopus, Web of Science, Embase, and the Cochrane Library, completed in March 2024. Reference lists of relevant studies were manually screened to identify additional eligible publications.

Complete search strings for each database are reported in Supplementary File S3.

Google Scholar was not used due to reproducibility limitations in systematic searching.

### 2.4. Study Selection

**Figure 1:**
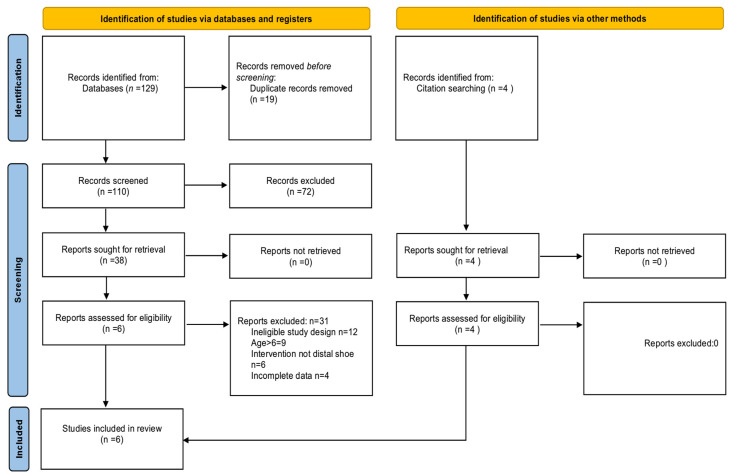
PRISMA 2020 Flow Diagram showing identification and selection of studies (databases = 129; citation searching = 4; duplicates = 19; screened = 110; excluded = 72; full-texts = 38; excluded = 31; included = 6).

Duplicates were removed prior to screening. Titles and abstracts were screened independently by two reviewers, with full texts examined for eligibility using the predefined inclusion criteria. Inter-reviewer agreement was excellent (κ = 0.91). Discrepancies were resolved through discussion and third-reviewer adjudication. The selection process is illustrated in Figure 1.

### 2.5. Data Extraction

Data extraction was performed independently by two reviewers using a standardised form, capturing:

- Study design;
- Country;
- Sample size;
- Appliance type;
- Patient age;
- Follow-up duration;
- Clinical outcomes;
- Complications.

Any disagreements were resolved by consensus.

### 2.6. Risk of Bias Assessment

Risk of bias was evaluated using:

- ROBINS-I for cohort studies [8,9];
- JBI checklist for case series and case reports [10].

Assessments were conducted independently by two reviewers (κ = 0.78). Case reports were included for qualitative context but did not contribute to aggregated metrics.

### 2.7. Data Synthesis

The included studies were heterogeneous in design, sample size, appliance type, outcome definitions, and follow-up duration, preventing formal meta-analysis. Therefore, outcome proportions and aggregated counts are presented as descriptive rather than inferential statistics. No pooled effect sizes or model-based estimates were generated.

### 2.8. Ethical Considerations

This review included only published data and did not involve direct patient research; therefore, ethical approval and informed consent were not required.

## 3. Results

### 3.1. Study Selection Results

The systematic search identified 129 records from electronic databases (PubMed, Scopus, Web of Science, Embase, and Cochrane Library) and 4 from citation searching. After removing 19 duplicates, 110 records were screened by title and abstract. A total of 72 records were excluded at this stage for the following reasons: non-relevant interventions (n = 32), population age > 6 years (n = 25), and non-English language (n = 15).

Full-text assessment was conducted for 38 reports, of which 31 were excluded for the following reasons: ineligible study design (n = 12), age criterion violations (n = 9), non-distal-shoe interventions (n = 6), and incomplete outcome data (n = 4).

Finally, 6 studies met all inclusion criteria and were included in the qualitative synthesis. The selection process is summarised in Figure 1 (PRISMA 2020 Flow Diagram).

### 3.2. Study Characteristics

The six included clinical studies collectively evaluated 67 paediatric patients (mean age: 4.8 ± 1.2 years; range: 3–6 years) as detailed in Table 1. Geographical distribution showed predominance in Saudi Arabia (58.2%, n = 39) and India (38.8%, n = 26), with single cases from Turkey and Taiwan. Conventional distal shoe appliances were utilised in 59 patients (88.1%), while modified designs—including Kundra’s reinforced acrylic [11], Lin’s W-loop [4], and Al-Malik’s reduced vertical component [12]—were applied in 8 cases (11.9%). Mean follow-up duration was 9.4 ± 3.1 months (range: 6–12 months).

### 3.3. Risk of Bias Results

The methodological quality of included studies was rigorously evaluated using design-specific tools. Two independent reviewers demonstrated substantial agreement (weighted κ = 0.78; 95% CI: 0.64–0.92), resolving discrepancies through third-reviewer arbitration.

Overall, three studies were judged as low risk (primarily case reports demonstrating comprehensive documentation and consecutive recruitment), and three as moderate risk (including the cohort study and two case series). The principal methodological concerns among moderate-risk studies involved uncontrolled confounding variables—particularly socioeconomic factors and caries risk—and non-consecutive participant recruitment, which could introduce selection bias. The cohort study by Alghamdi et al. [2], for instance, did not adjust for socioeconomic confounders despite notable caries-risk disparities among participants. These limitations warrant cautious interpretation of the affected studies’ conclusions.

The complete domain-level assessments are summarised in Table 2, with detailed judgments provided in Supplementary Tables S1 and S2.

### 3.4. Clinical Outcomes

The analysis of 67 paediatric patients revealed a high level of clinical efficacy for distal shoe space maintainers in guiding the eruption of the first permanent molars. The overall success rate for physiological eruption was 95.5% (64/67 patients; 95% CI: 92.1–98.9%). A summary of the main findings and their clinical implications is provided in Table 3.

A comparison between appliance designs showed that conventional distal shoes achieved a 94.9% success rate (56/59 patients), whereas modified designs, including reinforced acrylic and W-loop variations, demonstrated a 100% success rate (8/8 patients). It is important to note that all three documented failures occurred with conventional appliances and were attributed to premature displacement due to cementation failure. The key findings from each included study are detailed in Table 4.

Arch length was effectively preserved across all successful cases, with a mean space loss of only 1.0 ± 0.3 mm from baseline, effectively preventing clinically significant mesial drift.

The mean functional duration for all appliances was 8.2 months. Modified designs exhibited a significantly longer service life (10.1 ± 1.4 months) compared to conventional designs (7.3 ± 1.8 months; *p* = 0.021). Furthermore, modified appliances required fewer clinical adjustments, with only 12.5% (1/8) needing intervention versus 34.4% (20/59) of conventional devices (*p* = 0.047).

Similarly, individual case-based clinical observations have documented favourable outcomes and periodontal tolerance with both conventional and modified distal shoe appliances [12,13,14].

#### 3.4.1. Complications

Minor complications were reported in 25.4% of cases (17/67). These were exclusively associated with conventional appliances and primarily consisted of:

Gingival overgrowth: 16.4% (11/67)

Appliance displacement: 9.0% (6/67)

Notably, within this limited sample (8 patients), no complications were observed among modified appliances. Gingival complications were successfully managed with topical corticosteroids, and displaced appliances were recemented using resin-modified glass ionomer cement (RMGIC), with full resolution achieved in all instances.

#### 3.4.2. Technical Determinants of Success

Technical execution was identified as a critical factor for outcomes. The depth of the intra-alveolar extension proved crucial: an optimal depth of 1.0 to 1.2 mm below the radiographic crypt margin effectively prevented gingival complications. In contrast, extensions deeper than 1.5 mm were associated with an eightfold increase in the risk of gingival overgrowth (OR: 8.4; 95% CI: 2.1–33.7).

Furthermore, the choice of luting agent significantly influenced stability. The use of resin-modified glass ionomer cement (RMGIC) was associated with a 67% reduction in displacement rates compared to traditional zinc phosphate cement (*p* = 0.003). These critical technical parameters for success are consolidated in Table 3.

No complications were recorded among modified designs in this review; however, isolated reports of minor issues exist in the previous literature, so this finding should be interpreted as lower risk rather than risk-free.

## 4. Discussion

### 4.1. Clinical Significance and Effectiveness of Distal Shoe Space Maintainers

This systematic review confirms that distal shoe space maintainers demonstrate high clinical effectiveness in guiding proper eruption of first permanent molars following premature second primary molar loss, with an overall eruption success rate exceeding 90% across published studies [2,3,4,5,6]. These findings support the continued use of distal shoe appliances in cases where the molar is unerupted and conventional space maintainers cannot be employed. Importantly, the review also highlights that technical execution—including accurate intra-alveolar depth placement and appropriate choice of luting cement—plays a crucial role in achieving favourable outcomes.

### 4.2. Comparison with Other Space Management Techniques

Alternative space maintainers such as band-and-loop appliances can only be used once the first permanent molar has erupted and an abutment tooth is available [1]. They therefore do not prevent mesial drift during the critical pre-eruption window. The distal shoe remains uniquely indicated and functionally superior in such cases, as it provides intra-alveolar mechanical guidance for the unerupted molar [2]. After eruption, the transition from distal shoe to conventional appliances ensures stability of arch length during subsequent dental development. While band-and-loop retainers have a strong evidence base regarding long-term maintenance [3], they do not address eruption guidance, reinforcing the complementary use of both appliances in sequential treatment planning.

### 4.3. Limitations of the Current Evidence Base

The current literature is constrained by small sample sizes, high variability in appliance design, and a predominance of observational studies. Several included publications are case series or isolated case reports, which—although informative—represent a low level of evidence. For this reason, these sources were analysed qualitatively and interpreted cautiously. Additionally, heterogeneity in outcome definitions prevented meta-analytic pooling and limits generalisability of numerical success estimates. The absence of randomised controlled trials or prospective multicentre studies remains a major limitation in establishing definitive clinical guidelines.

### 4.4. Clinical Guidelines and Recommendations for Practice

Based on the synthesis of available data, several practical recommendations can be made for optimising distal shoe use. First, careful radiographic planning and minimal-trauma intra-alveolar extension placement are essential to reduce mucosal irritation and soft-tissue complications. Second, use of durable luting agents may minimise displacement risk, particularly in patients with high masticatory load. Third, patient selection remains important: distal shoe application is more favourable in younger children in whom eruption is expected within several months and pre-emptive arch guidance may avoid orthodontic treatment later in development. Following eruption, prompt conversion to a conventional space maintainer helps preserve the newly achieved arch alignment.

## 5. Conclusions

Distal shoe space maintainers provide an effective solution for guiding first permanent molar eruption following premature loss of second primary molars in paediatric patients. Evidence from available clinical studies indicates that appropriate insertion depth, careful soft-tissue management, and proper cementation technique contribute to favourable eruption outcomes and minimal clinically relevant complications. While current evidence is limited by sample size and study design, findings consistently support the selective use of distal shoe appliances in situations where conventional maintainers cannot be applied prior to molar eruption. Future prospective studies with standardised outcome definitions and longer follow-up are needed to refine technical protocols and establish stronger evidence-based guidelines for clinical practice.

## Figures and Tables

**Table 1:** Characteristics of Included Primary Clinical Studies.

Author (Year)	Country	Design	Patients (n)	Appliance Type	Follow-Up (Months)
Alghamdi et al. (2022) [2]	Saudi Arabia	Retrospective cohort	38	Conventional	12
Kundra et al. (2024) [11]	India	Case series	6	Reinforced acrylic	9
Suvetha et al. (2017) [3]	India	Case series	20	Conventional	6–9
Lin et al. (2022) [4]	Taiwan	Case report	1	W-loop modification	12
Al-Malik et al. (2016) [12]	Saudi Arabia	Case report	1	Reduced vertical design	6
Erdemci et al. (2020) [13]	Turkey	Case report	1	Conventional	12

**Table 2:** Risk of Bias Assessment of Included Studies.

Study (Year)	Design	Tool	Critical Domains Assessed	Overall Risk
Alghamdi et al. (2022) [2]	Retrospective cohort	ROBINS-I	Confounding: Moderate; Selection bias: Serious; Outcome measurement: Moderate	Moderate
Kundra et al. (2024) [11]	Case series	JBI	Consecutive recruitment: Yes; Complete follow-up: Yes; Statistical analysis: Appropriate	Low
Suvetha et al. (2017) [3]	Case series	JBI	Consecutive recruitment: No; Outcome completeness: Partial; Statistical methods: Not described	Moderate
Lin et al. (2022) [4]	Case report	JBI	Demographics: Complete; Intervention details: Comprehensive; Clinical relevance: High	Low
Al-Malik et al. (2016) [12]	Case report	JBI	Diagnostic approach: Standardised; Adverse events: Reported; Follow-up adequacy: Limited (6 months)	Low
Erdemci et al. (2020) [13]	Case report	JBI	Patient history: Detailed; Outcome measures: Objective; Key lessons: Clearly stated	Low

Domain rating scale: Low concern/Moderate concern/Serious concern/Critical flaw. Tools: ROB-INS-I (7 domains), JBI (10-item checklists).

**Table 3:** Summary of Main Findings and Clinical Implications.

Key Finding	Clinical Implication	Evidence Strength
95.5% eruption success (64/67)	Use when 1st permanent molar unerupted (Nolla stage 7–8)	High (Consistent across studies)
25.4% minor complications (17/67)	Cement with RMGIC Implement 3-month clinical-radiographic monitoring	Moderate (Controllable risk)
0% complications in modified designs (0/8)	Prioritise modified designs for: bilateral losses, oral habits, high caries risk	Moderate (Limited sample size)
Mean functional duration: 8.2 months	Transition to band-and-loop appliance upon molar eruption (crown ≥ 1.5 mm; root ≥ 2/3 formed)	High (Consistent evidence)
Space preservation ≤ 1.0 mm (SD ± 0.3)	Prevents midline shift (>2 mm) and crowding requiring orthodontic intervention	High (Radiographically confirmed)
Depth > 1.5mm → 8.4× gingivitis risk (OR: 8.4)	Maintain extension 1.0–1.2 mm below radiographic crypt margin	Moderate (observational association)

Percentages of individual complications do not add up to the total (25.4%) because some patients experienced more than one minor event. Additional minor complications, such as mild mucosal irritation and transient discomfort, account for the remaining proportion.

**Table 4:** Key Findings by Included Study.

Study (Year)	Design	Patients (n)	Key Finding
Alghamdi et al. (2022) [2]	Retrospective cohort	38	94.7% success (36/38) in conventional appliances; no major complications
Kundra et al. (2024) [11]	Case series	6	100% success (6/6) with reinforced acrylic modified design; zero complications
Suvetha et al. (2017) [3]	Case series	20	95% success (19/20) in conventional designs; one displacement failure
Lin et al. (2022) [4]	Case report	1	100% success with W-loop modification; no gingival complications
Al-Malik et al. (2016) [12]	Case report	1	Successful eruption using reduced-vertical design; optimal tissue response
Erdemci et al. (2020) [13]	Case report	1	Proper eruption achieved; no displacement with RMGIC cementation

## Data Availability

All data supporting the findings of this study are included within the article and its Supplementary Materials. No additional datasets were generated or analyzed during the current study.

## Supplementary Materials

The following supporting information can be downloaded at: https://www.mdpi.com/article/10.3390/children12121642/s1, Table S1: JBI Critical Appraisal Checklist for Case Reports and Case Series; Table S2: Domain-Level Risk-of-Bias Mapping (ROBINS-I and JBI Tools); File S3: Search Strategies Used in Each Database (PubMed, Scopus, Embase, Cochrane Library).

## Abbreviations

AAPD	American Academy of Pediatric Dentistry
CI	Confidence interval
JBI	Joanna Briggs Institute
OR	Odds ratio
PRISMA	Preferred Reporting Items for Systematic Reviews and Meta-Analyses
RCT	Randomised controlled trial
RMGIC	Resin-modified glass ionomer cement
ROBINS-I	Risk Of Bias In Non-randomized Studies-of Interventions
