# Supplementary material for: Effectiveness of Distal Shoe Space Maintainers for First Permanent Molar Eruption: A Systematic Review
Source: Children (Basel). 2025 Dec 3;12(12):1642. doi: 10.3390/children12121642 (PMC12731900; doi:10.3390/children12121642)
Supplement: Supplementary file 1 [file children-12-01642-s001.zip › children-4001285-supplementary.pdf]

Supplementary Materials

Table S1. JBI Critical Appraisal Checklist for Case Reports and Case Series.

Study	Patient Demographics Clearly Described	Clinical Condition Reported in Detail	Diagnostic Tests Described	Intervention/Procedure Clearly Described	Outcomes Clearly Reported	Adverse Events Reported	Follow-up Adequate	Consecutive Recruitment	Statistical Analysis Appropriate	Overall Appraisal
Alghamdi et al., 2022	Yes	Yes	Yes	Yes	Yes	Yes	Yes	Yes	Yes	Low risk
Kundra et al., 2024	Yes	Yes	Yes	Yes	Yes	No (none observed)	Yes	Yes	Yes	Low risk
Suvetha et al., 2017	Yes	Yes	Partial	Yes	Yes	Partial	Yes	No	No	Moderate risk
Lin et al., 2022	Yes	Yes	Yes	Yes	Yes	No (none observed)	Yes	—	—	Low risk
Al-Malik et al., 2016	Yes	Yes	Yes	Yes	Yes	Yes	Partial	—	—	Low risk
Erdemci et al., 2020	Yes	Yes	Yes	Yes	Yes	No (none observed)	Yes	—	—	Low risk

Table S2. Domain-Level Risk-of-Bias Mapping (ROBINS-I and JBI Tools).

Study	Bias due to Confounding	Selection Bias	Classification of Interventions	Deviations from Intended Interventions	Missing Data	Measurement of Outcomes	Selection of Reported Result	Overall Risk
Alghamdi et al., 2022	Moderate	Serious	Low	Low	Low	Moderate	Low	Moderate
Kundra et al., 2024	Low	Low	Low	Low	Low	Low	Low	Low
Suvetha et al., 2017	Moderate	Moderate	Low	Low	Low	Moderate	Low	Moderate
Lin et al., 2022	Low	Low	Low	Low	Low	Low	Low	Low
Al-Malik et al., 2016	Low	Low	Low	Low	Low	Low	Low	Low
Erdemci et al., 2020	Low	Low	Low	Low	Low	Low	Low	Low

File S3. Search Strategies Used in Each Database

PubMed Search Strategy:

("distal shoe"[Title/Abstract] OR "intra-alveolar appliance"[Title/Abstract])
AND ("space maintainer"[Title/Abstract] OR "space management"[Title/Abstract])
AND ("premature tooth loss"[Mesh] OR "primary molar loss"[Title/Abstract] OR "early exfoliation"[Title/Abstract])
AND ("eruption guidance"[Title/Abstract] OR "molar eruption"[Title/Abstract])
AND (child[Title/Abstract] OR pediatric[Title/Abstract] OR paediatric*[Title/Abstract])
NOT (adult[Mesh] OR animals[Mesh])
Filters: English; Publication Date 1990/01/01–2024/03/31

Scopus Search Strategy:

TITLE-ABS-KEY("distal shoe" OR "intra-alveolar appliance")
AND TITLE-ABS-KEY("space maintainer" OR "space management")
AND TITLE-ABS-KEY("primary molar" OR "premature tooth loss")
AND TITLE-ABS-KEY("eruption guidance" OR "molar eruption")
AND TITLE-ABS-KEY(child OR pediatric OR paediatric)
AND NOT TITLE-ABS-KEY(adult)
LIMIT-TO(LANGUAGE, "English")

Embase Search Strategy:

('distal shoe':ab,ti OR 'intra alveolar appliance':ab,ti)
AND ('space maintainer':ab,ti OR 'space management':ab,ti)
AND ('primary molar loss':ab,ti OR 'early exfoliation':ab,ti)
AND ('eruption guidance':ab,ti OR 'molar eruption':ab,ti)
AND [english]/lim AND [1990-2024]/py
NOT [animals]/lim

Cochrane Library Search Strategy:

(distal shoe OR intra-alveolar appliance)
AND (space maintainer OR space management)
AND (primary molar OR premature tooth loss)
AND (eruption guidance OR molar eruption)
AND (child OR pediatric OR paediatric)
